# A National Big Data Analysis on Alzheimer’s and Other Dementias in Türkiye

**DOI:** 10.1155/jare/1010110

**Published:** 2026-02-02

**Authors:** Talip Yi̇ği̇t, Murat Di̇nçer, Naim Ata, M. Mahir Ülgü, Şuayip Bi̇ri̇nci̇, M. Okan Ayvali

**Affiliations:** ^1^ Faculty of Economics and Administrative Sciences, Istanbul 29 Mayıs University, İstanbul, Türkiye; ^2^ General Directorate of Health Information System, Turkish Ministry of Health, Ankara, Türkiye, saglik.gov.tr; ^3^ Counselor, Turkish Ministry of Health, Ankara, Türkiye, saglik.gov.tr; ^4^ Vice Minister, Turkish Ministry of Health, Ankara, Türkiye, saglik.gov.tr; ^5^ Computer Engineer, Independent Researcher, Ankara, Türkiye

**Keywords:** Alzheimer’s disease, dementia, logistic regression

## Abstract

**Introduction:**

Scientific and technological advances are emphasizing biomarker‐driven studies to diagnose, classify, and predict the pathogenesis of Alzheimer’s disease (AD) and dementias (Ds) with the development of intelligent systems during these symptom‐free years.

**Methods:**

In this study, the data of people over 65 who were diagnosed with AD/D in 2020 were retrospectively scanned, and the data of 243,073 people identified were analyzed on a total of 32 variables, 6 of which were dependent variables and 26 were independent variables.

**Results:**

It is understood from the logistic regression (LR) models that AD/D is a multilayered and extraordinarily complex condition with biological, mental, and social dimensions rather than a single variable or parameter.

**Discussion:**

As a main result of the study, it is understood that instead of generalized diagnostic criteria, the appearance and boundaries of AD in society can be determined with specialized technology‐based smart systems.

## 1. Introduction

The prevalence of dementia (D) is increasing day by day. D is an umbrella term that encompasses diseases resulting from damage to the brain, negatively affecting memory, thinking, and behavior [1]. As a result of this damage, individuals experience a faster decline in mental and cognitive levels compared to those experiencing normal aging processes [2, 3].

Among these diseases, Alzheimer’s disease (AD), discovered by Alois Alzheimer in 1906, is the most common, constituting approximately 60%–70% of D cases [1, 4]. The rate of AD per 100,000 people was 370 in 1990 and increased to 667 in 2019. This rate is 854 for women and 482 for men. Moreover, between 1990 and 2019, women have had a higher D rate compared to men every year [5]. With this prevalence, AD is the 17th most common cause of death in the United States [1, 6]. It is estimated that the total cost, which is currently approximately 1.3 trillion U.S. dollars [1], will reach 9.1 trillion U.S. dollars by 2050 due to AD [7].

Alzheimer’s initial symptoms can often be confused with the normal aging process [8, 9]. In the initial stages, there is a clear tendency to forget recent experiences and a weakening of abstract thinking skills [10]. In the middle stages, difficulties in speech, aggression, incontinence, crying behavior, and the need for institutional care can be observed [11, 12]. In the final stages of Alzheimer’s patients, there is a high level of apathy and exhaustion, leading to complete dependency on caregivers [11, 13].

Currently, AD, for which there is no definitive cure but is attempted to be controlled with drugs such as galantamine, rivastigmine, and donepezil, is a subject of focus in contemporary studies, with debates revolving around its preventability and/or delayability, estimated to be at 40% [14, 15]. However, the etiology of AD, which can be detected through techniques such as PET, MRI, and analysis of cerebrospinal fluid (CSF), is still not fully understood [16, 17]. Particularly, factors such as age, gender, and genetic factors (especially for diagnoses made before the age of 65) are considered immutable factors [18, 19]. Modifiable factors typically include a wide range of factors such as smoking and alcohol consumption, regular exercise, depression, diabetes, hypertension, head trauma, and other internal diseases [15, 20].

It is known that apathy and depression can be observed in the initial stages of AD [21, 22]. Especially, depression experienced in middle age has been shown to increase the risk of developing Alzheimer’s and other D diseases [23–26]. In addition, loneliness has been linked to rapid cognitive function loss associated with D diseases [27–29]. Furthermore, evidence exists indicating that organ failures, especially kidney failure, increase the risk of biochemical conditions associated with AD [30–32]. Moreover, hyperlipidemia [33, 34], hypertension, and diabetes, particularly in middle age, are known risk factors for D [14, 35]. Old age associated with D is a widespread phenomenon worldwide. The proportions of the population aged 65 and over are estimated to be 16.4% and 25.6% for the world and Türkiye, respectively, in 2050 [36, 37].

Therefore, the aim of the study is to contribute to studies focusing on technical analyses related to D diseases, especially AD, in the preclinical process. Thus, the study includes models aiming to predict the relationship between preclinical illnesses and AD/D. To achieve this goal, data from all individuals who underwent medical inquiries related to AD/D in the Türkiye in 2020 have been analyzed. Based on strong evidence from the literature regarding the relationship between chronic illnesses and AD/D, observations on chronic illness diagnosis were used to compare AD/D diagnosis with its prevalence [38, 39].

## 2. Methods

This study was conducted with the permission of the Ministry of Health of the Republic of Türkiye. Within the scope of the study, the data of a total of 243,073 people who were questioned for D in health institutions within the borders of the Republic of Türkiye in 2020 were retrieved by the e‐nabız​ system developed by the Ministry of Health of the Republic of Türkiye. In the original dataset, there were 560 more people (0.23%) in the dataset with at least one observation missing, since their small proportion in the total listwise deletion method is used for missingness. The examinations were conducted on two separate datasets, one consisting of individuals diagnosed and undiagnosed with AD, and the other consisting of individuals diagnosed and undiagnosed with D. The analyses were performed using logistic regression (LR) through the *R* Studio 4.2.0 program. The codes for LR analysis are given in Annex 1.

Transformed odds ratios were used in the interpretation of LR models as a classification method. Therefore, the dataset was divided into 70–30 and 80–20 splits, and two different models were built for each variable group. Ideal models were decided based on accuracy, Akaike information criterion (AIC), sensitivity, and specificity values.

Annex 2 has explanations for the variables in the dataset. There are a total of 35 variables in the dataset, including 6 dependent and 26 independent variables. Considering the possibility of increasing the error rate of the model due to the considerable number of independent variables, the independent variables were divided into 4 distinct groups. In addition, the Pearson correlation coefficient between the variables in each variable group was calculated before the analysis to evaluate the assumption of no correlation greater than 0.90 among the independent variables (multicollinearity). According to the results, the variables “intensive care unit” and “geriatrics” within the “diagnosis” variable group showed a correlation coefficient higher than 0.90 with each other and with other variables. Therefore, coefficients for these variables were calculated separately in the models.

In total, 72 different LR models were built based on these criteria. After excluding highly correlated variables, coefficients with *p* value greater than 0.05 were also excluded from the model, and coefficients were recalculated. In addition, the variables “die.month,” “intensive care unit,” and “hospitalize” were converted into categorical variables due to the imbalanced distribution of observations for these variables, aiming to increase the statistical significance of the results. Annex 3 shows the numerical distributions of numerous factors in the dataset. Analyses were conducted to illustrate the relationship between dependent variables and independent variables.

## 3. Results

Table 1 contains a summary of the ideal LR model results. Information on which of the 70%–30%/80%–20% models is the ideal model for each analysis based on variable group is presented in Annex 4.

**Table 1:** Coefficients of the LR models (%)∗.

Independent variables	Alzheimer’s disease/dementia		Dementia medicines		Lifetime	
	Alzheimer’s disease	Dementia	Alzheimer’s disease	Dementia	Alzheimer’s disease	Dementia
Sex [2]	10.9	8.15	!	!	−13.8	−15.25
Age	1.47	0.56	−0.96	−0.72	1.51	1.01
Foreigner [2]	49.11	!	!	!	!	!
Social security [2]	4.57	!	!	!	!	!
Social security [3]	7.06	!	!	!	!	!
Intens. care unit [2]	76.36	41.21	!	!	1.05	54.74
Intens. care unit [3]	1.89	1.19	!	!	1.31	90.54
Hospitalize [2]	6.26	5.05	!	!	13.22	12.54
Hospitalize [3]	33.35	32.21	!	!	56.77	43.52
Internal diseases [2]	35.19	37.01	17.08	19.27	!	!
Geriatrics [2]	37.46	37.46	17.87	19.49	!	!
Neurology [2]	6.19	5.96	1.3	1.46	−51.5	−52.89
Psychiatry [2]	−25.9	−16.2	−24.1	−15.61	!	6.36
Emergency [2]	−11.7	−19.1	−27.9	−29.54	23.7	26.05
Prerenal failure [2]	−0.02	−0.02	−0.02	−0.02	−0.02	0.02
Preliver failure [2]	−0.03	!	−0.06	!	!	!
Preheart failure [2]	0	−0.01	−0.02	−0.02	−0.02	0.02
Preaf [2]	−0.01	−0.01	−0.01	−0.01	−0.01	0.01
Precerobro [2]	−0.01	−0.02	−0.01	−0.02	!	0.01
Precoronary [2]	0	−0.03	−0.02	−0.03	!	0.02
Prediabetes [2]	−0.01	−0.01	0	−0.01	!	!
Prehyperlipidemia [2]	−0.01	−0.01	0	!	−0.06	!
Prehypertension [2]	−0.06	−0.05	−0.04	−0.05	−0.02	−0.05
Precopd [2]	−0.02	−0.01	−0.02	−0.02	−0.01	−0.02
Preosteoporosis [2]	−0.02	−0.01	−0.01	−0.01	!	!
Prepsychological [2]	−0.01	−0.01	−0.01	−0.01	−0.01	!

^∗∗^The coefficients presented are statistically significant at least at 95% significance level (∗). The coefficients for the variables marked “!” are not statistically significant at 95% significance level in the models established with the relevant dependent variable.

The numerical values in Table 1 show the percentage of positive or negative impact each variable has on the relevant dependent variable. For example, having a gender value of 1 (*female*) instead of 0 (*male*) increases the probability of being diagnosed with AD by 10.9%.

The results obtained in the LR models show that being a foreigner and being in different social security categories do not have any effect on D but have a positive effect on Alzheimer. Being a woman increases the likelihood of diagnosis and shortens life expectancy. Being diagnosed in internal disease and geriatric clinics and the increase in the “intensive care unit” and “hospitalize” variables have a moderate or higher effect on diagnosis. However, the coefficient is negative for the variables “psychiatry” and “emergence” in the same category, and it shows a decreasing effect on the dependent variables. In addition, it was seen that all prediseases either had no effect or had a minimal negative effect on all dependent variables.

**Figure 1:**
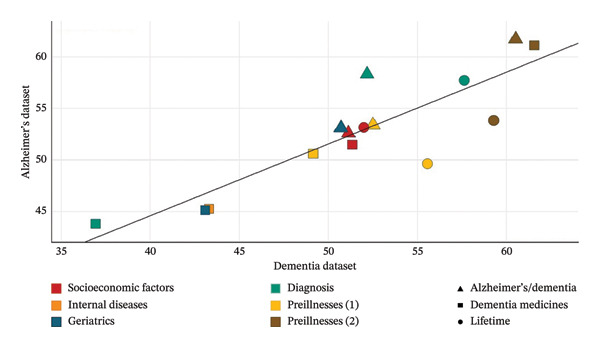
Accuracy rates of LR models.

The correct prediction rates of the LR models obtained within the scope of the study vary between 36.94% and 61.72%. In addition, it was seen that there were significant differences between selectivity and sensitivity values in some models. In addition, the average correct prediction rate is 52.75% for models established with the Alzheimer’s dataset and 51.75% for the models established with the D dataset. Figure 1 shows the distribution of correct prediction rates of all established LR models. As can be seen from Figure 1, the distribution of correct prediction rates is concentrated in the range of 40%–60%. Accuracy values of each model are shown in Annex 5.

This shows that the established LR models only have a moderate prediction success. In other words, the variables in the dataset do not have sufficient explanatory power to explain the AD/D situation. These results show that individuals’ social and biological factors have insufficient explanatory power to explain the AD/D diagnosis situation, and that these diseases should be evaluated from a holistic perspective. In other words, the inability of numerous factors to explain AD/D alone has highlighted the necessity of examining the factors that cause these diseases in a holistic manner.

## 4. Discussion

The recent discovery that AD is initiated by tau protein accumulation 20 years or more before the onset of symptoms suggests that there is an important period in which we can intervene in disease progression. With the development of intelligent systems in these symptom‐free years, scientific advances are emphasizing computer‐assisted diagnosis, classification of AD, and biomarker‐driven studies to predict the pathogenesis of AD. However, various findings indicating that the course of the disease is characterized by a slowdown in the rate of cerebral metabolism 10–15 years before the onset of symptoms and cognitive impairment up to 5 years before the onset of symptoms, while patients can fulfill the diagnostic criteria only 3 years after the onset of symptoms, reveal the importance of these biomarker‐oriented studies [40–42].

Similarly, in the last 10 years, there has been an exponential increase in the number of scientific studies on data analytics of large patient samples, as well as the use of neural networks to analyze complex datasets. Innovative solutions are becoming an increasing part of neurology and medicine in general. Big data–based modeling, especially for technical analyses regarding the diagnosis and treatment of people with AD/D in the preclinical process, is important in terms of showing in which life patterns the disease develops. This study is designed to contribute to such a strategic goal.

The results obtained within the scope of the study show that there are different patterns for AD/D. Although some of the independent variables that have a high level of impact on the relevant dependent variables in LR models provide important clues about the general well‐being of people with AD, when considered together with other independent variables, LR models have only moderate explanatory power regarding the etiology and protogenesis of AD. These results suggest that many basic assumptions about AD/D should be evaluated from a unique perspective.

In the study, some remarkable findings were found for this necessity. For example, it was found that a one‐unit increase in a person’s age increases the probability of being diagnosed with AD by 1.47%, while it increases only 0.56% for D diseases. These findings reveal that, contrary to widespread belief, the assumption that AD/D are diseases of old age should be reexamined. This is because the conditions that cause AD are most likely to be seen above the age of 65, which is related to the change in the life pattern of the person, more specifically the level of social and cognitive interaction. What is clear is that staying socially and mentally active throughout life supports brain health and reduces the risk of AD and other Ds [43]. Naturally, the conditions that lead to AD can develop independently of age periods. This is supported by research on genetic susceptibility between AD and aging. With the advent of next‐generation sequencing and the increase in genomewide association studies, more than 20 risk loci for AD have been identified, but not including the (FOXO3) mutation, which is known to have a strong impact on aging and aging‐related phenotypes [44]. It seems that if certain conditions occur, AD can inevitably develop independently of aging, and the assumed epigenetic programming associated with AD becomes active. Therefore, while the life pattern experienced after the age of 65 currently constitutes the basic conditions for Alzheimer’s pathogenesis, if these conditions occur earlier, it will imply the occurrence of AD at much younger ages. So, the main thing to understand is the conditions under which the epigenetic software of AD develops. Indeed, the diagnosis of AD in a 19‐year‐old male, despite the absence of any genetic mutations, has shown that this young age AD condition, previously defined as early‐onset AD and largely associated with genetic factors, can occur independently of a genetic basis [45]. Based on this, it is thought that aging may not be a determinant factor for AD but can be considered as a descriptive variable.

Another significant finding obtained in the research is that the probability of being diagnosed with AD increases by 49.11% for people who are not citizens of the Republic of Türkiye. This result is interpreted to mean that migration and foreign experience involve an incomparably higher risk probability for AD diagnosis, especially compared to old age, which is accepted as a basic assumption. Similarly, many studies have shown that belonging to a different ethnicity in the current society increases the risk of AD [46–48]. However, an in‐depth understanding of the meaning of these research results is important for the perspective of this study, since all these research findings suggest that AD is a multilayered condition with biological, mental, and social dimensions, and not a single variable or parameter. Therefore, the fact that a person’s ethnic identity differs in the community in which they are present is, in a sense, a summary of a complex and multifaceted life pattern, such as AD. Hence, among the aging‐related variables that are assumed to be the main risk factor of AD, the fact that ethnic identity difference has a much higher risk possibility is related to the fact that it fully represents the life pattern.

In summary, understanding the pathogenesis of AD depends on identifying how and to what extent an individual’s life pattern interacts with it. Consequently, rather than relying on traditional symptomatic diagnostic criteria for AD, the use of highly specialized intelligent systems can help delineate the appearance and boundaries of AD in society. With the ability to predict the personalized pathogenic process of AD, innovative, data‐driven interventions can be developed for prophylactic measures. Moreover, biomarker‐oriented discoveries that AD symptoms started 20 years ago can be intervened in the context of truly person‐centered medicine, and the conditions under which the epigenetic software of AD works can be determined in the preclinical process.

### 4.1. Strengths and Limitations of the Study

This study has some limitations and strengths based on its retrospective and observational nature. First, the quality of observations and the power of the data in reflecting real‐life conditions totally depend on administrative processes. In addition, other important factors that could be related with AD/D experiences, such as education and income, are not presented totally in the dataset. Besides, the models built in this study only achieved moderate statistical power. Furthermore, the data reflect a single year and one country, which may be a limit to understanding the situation itself.

## Author Contributions

Naim Ata conducted a literature review and contributed to the analyses. Talip Yi̇ği̇t and Murat Di̇nçer mostly worked on statistical analysis and data preparation. Naim Ata handled data extraction and official processes on it. Şuayip Bi̇ri̇nci̇ contributed to the introduction and conclusion. M. Mahir Ülgü, Şuayip Bi̇ri̇nci̇, and M. Okan Ayvali participated in conceptualizing and result interpretation processes.

## Funding

The authors received no financial support for the research, authorship, and/or publication of this article.

## Disclosure

All authors approved the final version of the manuscript.

## Consent

Data on Alzheimer’s and other dementia diseases were obtained through the “e‐nabız” system, which is the national health database. The system is open to all citizens across the country. The transactions conducted by people in health institutions are recorded in that person’s profile. This registration is carried out on the basis of the principles within the scope of both the “Personal Data Protection Law” No. 6698 and the “Law on the Establishment of the Ethics Committee for Public Servants” No. 5176, which regulates the work of the Ethics Commission of the Ministry of Health. In the system, personal data are preserved and archived by the Ministry and not shared with third parties. For all these reasons, the Ministry of Health, as the most authorized institution, does not have an organization that would approve the use of these data on a confidential basis. Under these conditions, informed consent was waived by the Ministry of Health, and since the Ministry already has the official power to collect, store, and use these data, another official permission is not necessary in this context.

## Conflicts of Interest

The authors declare no conflicts of interest.

## Supporting Information

Supporting 1. 4. Research in context.

Supporting 2. This document summarizes the literature search strategy, key interpretations of the study findings, and outlines future directions to contextualize the results within existing Alzheimer’s disease research.

## Supporting information


**Supporting Information** Additional supporting information can be found online in the Supporting Information section.

## Data Availability

The data that support the findings of this study are available from the Republic of Türkiye Ministry of Health, but restrictions apply to the availability of these data, which were used under license for the current study, and so are not publicly available. Data are, however, available from the authors upon reasonable request and with permission of the Republic of Türkiye Ministry of Health. Apart from personal data, data related to diseases and treatments applied are used in studies conducted by Ministry researchers. This authority is guaranteed by the Ministry’s establishment laws and relevant legislation. Moreover, since the Ministry already has the official power to collect, store, and use these data, another official permission is not necessary in this context.
