# Supplementary material for: A National Big Data Analysis on Alzheimer’s and Other Dementias in Türkiye
Source: J Aging Res. 2026 Feb 2;2026:1010110. doi: 10.1155/jare/1010110 (PMC12864165; doi:10.1155/jare/1010110)
Supplement: Supplementary file 1 — Supporting Information Additional supporting information can be found online in the Supporting Information section. [file JARE-2026-1010110-s001.zip › 2.Annexes.docx]

**Annex 1: R Studio Codes for LR Analysis**

install.packages("readxl")

install.packages("Rcpp")

install.packages("tidyverse")

install.packages("caret")

install.packages("stats")

install.packages("caTools")

install.packages("psych")

library(readxl)

library(Rcpp)

library(tidyverse)

library(caret)

library(stats)

library(caTools)

library(psych)

data1 <- alz[,5]

data2 <- alz[,11]

data <- cbind(data1,data2)

summary(data)

outliers <- function(x) {

Q1 <- quantile(x, probs=.25)

Q3 <- quantile(x, probs=.75)

iqr = Q3-Q1

upper_limit = Q3 + (iqr*1.5)

lower_limit = Q1 - (iqr*1.5)

x > upper_limit | x < lower_limit

}

remove_outliers <- function(df, cols = names(df)) {

for (col in cols) {

df <- df[!outliers(df[[col]]),]

}

df

}

summary(df)

df_temiz <- remove_outliers(df, c('bmi',

'yas',

'yb',

'servis',

't.tani',

't.hastalik',

'pre.hastalik',

'post.hastalik'))

df_temiz <- remove_outliers(df, c('yas'))

summary(df_temiz)

str(df_temiz)

df <- df_temiz

df$alz <- as.factor(df$alz)

df$i.demans <- as.factor(df$i.demans)

df$olum.ay <- as.factor(df$olum.ay)

df$t.basvuru <- as.factor(df$t.basvuru)

df$cinsiyet <- as.factor(df$cinsiyet)

df$yabanci <- as.factor(df$yabanci)

df$sigorta <- as.factor(df$sigorta)

df$ic.hst <- as.factor(df$ic.hst)

df$geriatri <- as.factor(df$geriatri)

df$noroloji <- as.factor(df$noroloji)

df$ruh.sglk <- as.factor(df$ruh.sglk)

df$acil.tip <- as.factor(df$acil.tip)

df$kalp.ytmz <- as.factor(df$kalp.ytmz)

df$af <- as.factor(df$af)

df$karaciger.hst <- as.factor(df$karaciger.hst)

df$solid.knsr <- as.factor(df$solid.knsr)

df$hmt.knsr <- as.factor(df$hmt.knsr)

df$depresyon <- as.factor(df$depresyon)

df$osteoporoz <- as.factor(df$osteoporoz)

df$kalca.krk <- as.factor(df$kalca.krk)

df$pre.diyabet <- as.factor(df$pre.diyabet)

df$pre.serobro <- as.factor(df$pre.serobro)

df$pre.hiperlipidemi <- as.factor(df$pre.hiperlipidemi)

df$pre.koah <- as.factor(df$pre.koah)

df$pre.hipertansiyon <- as.factor(df$pre.hipertansiyon)

df$pre.bobrek.ytmz <- as.factor(df$pre.bobrek.ytmz)

df$pre.koroner <- as.factor(df$pre.koroner)

df$post.diyabet <- as.factor(df$post.diyabet)

df$post.serobro <- as.factor(df$post.serobro)

df$post.hiperlipidemi <- as.factor(df$post.hiperlipidemi)

df$post.koah <- as.factor(df$post.koah)

df$post.hipertansiyon <- as.factor(df$post.hipertansiyon)

df$post.bobrek.ytmz <- as.factor(df$post.bobrek.ytmz)

df$post.koroner <- as.factor(df$post.koroner)

set.seed(123)

df_2 = sort(sample(nrow(df), nrow(df)*.7))

df_train<- df[df_2,]

df_test<- df[-df_2,]

summary(df_train)

summary(df_test)

summary(df)

set.seed(111)

traindown<-downSample(x=df_train,

y=df_train$alz)

summary(traindown)

table(traindown$alz)

set.seed(123)

summary(traindown)

modeldown <- glm(alz~relevel(cinsiyet,ref="0")+

yas+

relevel(yabanci,ref="0")+

relevel(sigorta,ref="0"),

data=traindown, family="binomial")

summary(modeldown)

pred <- predict(modeldown,df_test, type="response")

pred <- as.integer(pred>0.5)

confusionMatrix(as.factor(pred),df_test$alz)

(exp(modeldown$coefficients[-1])-1)

**Annex 2: Explanations on Variables**

| Variable  Group | Name | Type | Explanation |
| --- | --- | --- | --- |
| Dependent Variables | Alzheimer’s | Binary | Whether the person has been diagnosed with Alzheimer's  *Only in the "alzheimer's" dataset  (ICD 10 Codes: F00.0, F00.1, F00.2, F00.9 or G30) |
|  | dementia | Binary | Whether the person has been diagnosed with Dementia  *Only in the "dementia" dataset  (ICD 10 Codes: F01, F02, F02.0, F02.1, F02.2, F02.3, F02.4, F02.8, F03 or G31 ) |
|  | Dementia.medicine | Binary | Whether the person has used at least one of the; donepezil”, “donepezil.memantin”, “galantamin”, gingko”, “memantin”, “rivastigmin” medicine |
|  | Life.time | Binary | Total time of life after alzheimer’s/dementia questioning  (<12 months “1”, >12 months “0”) |
| Socioeconomic Factors | sex | Binary | Sex of the person (0: Male 1: Female) |
|  | age | Contunious | Age of the person |
|  | foreigner | Binary | Whether the person has citizenship of the Republic of Türkiye (0: Yes 1: No) |
|  | Social.security | Ordinal | Registered social security system   (0: nonexistence, 1: General Health Insurance [GSS]  2: Others [Bağkur, Retirement Fund, Social Security Insurance (SGK) etc.]) |
| Diagnosis | Intensive.care.unit (ICU) | Ordinal | Whether the person has been treated in intensive care units  (“0”: not, “1”. Treated along 1-5 days, “2”: Treated more than 5 days) |
|  | Hospitalize | Ordinal | Whether the person has been treated in hospita services except intensive care units  (“0”: not, “1”. Treated along 1-5 days, “2”: Treated more than 5 days) |
|  | Internal.diseases | Binary | Whether the person has been diagnosed by internal diseases departments |
|  | geriatrics | Binary | Whether the person has been diagnosed by geriatrics departments |
|  | neurology | Binary | Whether the person has been diagnosed by neurology departments |
|  | psychiatry | Binary | Whether the person has been diagnosed by psychiatry departments |
|  | emergency | Binary | Whether the person has been diagnosed by emergency medicine departments |
| Pre Illnesses (1)  *before the Alzheimer's questioning | pre.renal.failure | Binary | Whether the person was diagnosed with renal failure  (ICD 10: N14/N19) |
|  | pre.liver.failure | Binary | Whether the person was diagnosed with liver failure (ICD 10: K72) |
|  | pre.heart.failure | Binary | Whether the person was diagnosed with heart failure (ICD 10: I50) |
|  | pre.af | Binary | Whether the person was diagnosed with atrial fibrillation (ICD 10: I48) |
|  | pre.cerebrovascular | Binary | Whether the person was diagnosed with cerebrovascular accident  (ICD 10: I64) |
|  | pre.coronary | Binary | Whether the person was diagnosed with coronary artery (ICD 10: I25.1) |
| Pre Illnesses (2)  *before the Alzheimer's questioning | pre.diabetes | Binary | Whether the person was diagnosed with hiabetes mellitus (ICD 10: E10/E14) |
|  | pre.hyperlipidemia | Binary | Whether the person was diagnosed with hyperlipidaemia (ICD 10: E78.2/E78.4/E78.5) |
|  | pre.hypertension | Binary | Whether the person was diagnosed with hypertension (ICD 10: I10/I15) |
|  | pre.copd | Binary | Whether the person was diagnosed with Chronic Obstructive Pulmonary Disease  (ICD 10: J44.9) |
|  | pre.osteoporosis | Binary | Whether the person was diagnosed with osteoporosis (ICD 10: M80/M81/M82) |
|  | pre.psy | Binary | Whether the person was diagnosed with psychological disorders, in particular depression and bipolar disorder, (ICD 10: F31/F32/F33/F34.1/F06.3) |

***Binary:** Binary categorical variable (0-1) **/** **Ordinal:** Variable with more than two hierarchical categories

** All of the condings in the same way as “yes = 1” and “no = 0”

**Annex 3 Summary Information on the Data Set**

| Category | Observation  Number | Rate of  m.dementia  use (%) | Sex (%) |
| --- | --- | --- | --- |
| Total | 243,073 | 59.7 | Male: 39.09  Female: 60.91 |
| Alzheimer’s | 88,157 | 63.07 | Male: 37.71  Female: 62.29 |
| Dementia | 62,196 | 54.92 | Male: 38.73  Female:61.27 |
| Non-Diagnosed | 92,720 | --- | Male: 40.65  Female: 59.35 |
| Category | **Average**  **Age**  **(min-max)** | **Mortality**  **Rate (%)** | **Average**  **Time of Life**  **After**  **Alzheimer/Dementia**  **Diagnosis (month)** |
| Total | 79.22  (65-132) | 46.47 | 15.87 |
| Alzheimer’s | 79.7  (65-132) | 49.23 | 17.82 |
| Dementia | 79.17  (65-118) | 46.56 | 18.37 |
| Non-Diagnosed | 78.81 (65-125) | 43.77 | 11.99 |

**Annex 4: Selected Ideal Models of LR Analysis**

| Alzheimer Dataset | | | |
| --- | --- | --- | --- |
| Independent Variable Group | **Dependent Variable** | | |
|  | **AD/D** | **Dementia Medicine** | **Life Time** |
| Socioeconomic Factors | %70-%30 | %70-%30 | %80-%20 |
| Internal diseases | %70-%30 | %70-%30 | *** |
| Geriatrics | %70-%30 | %70-%30 | *** |
| Diagnosis | %70-%30 | %70-%30 | %70-%30 |
| Pre Illnesses (1) | %80-%20 | %70-%30 | %70-%30 |
| Pre Illnesses (2) | %70-%30 | %70-%30 | %70-%30 |
| Dementia Dataset | | | |
| Independent Variable Group | **Dependent Variable** | | |
|  | **AD/D** | **Dementia Medicine** | **Life Time** |
| Socioeconomic Factors | %70-%30 | %70-%30 | %70-%30 |
| Internal diseases | %70-%30 | %70-%30 | *** |
| Geriatrics | %70-%30 | %70-%30 | *** |
| Diagnosis | %70-%30 | %70-%30 | %70-%30 |
| Pre Illnesses (1) | %70-%30 | %70-%30 | %70-%30 |
| Pre Illnesses (2) | %80-%20 | %70-%30 | %80-%20 |

***A statistically significant model could not be developed.

**Annex 5: Accuracy Values of LR Models**

| Dependent Variable | Independent Variable | Alzheimer | Dementia |
| --- | --- | --- | --- |
| ad/dm | Socioeconomic Factors | 52.61 | 51.13 |
| ad/dm | internal.affairs | 53.11 | 50.71 |
| ad/dm | geriatrics | 53.11 | 50.71 |
| ad/dm | Diagnosis | 58.33 | 52.18 |
| ad/dm | Pre Illnesses (1) | 53.39 | 52.51 |
| ad/dm | Pre Illnesses (2) | 61.72 | 60.51 |
| m.dm | Socioeconomic Factors | 51.49 | 51.34 |
| m.dm | internal.affairs | 45.27 | 43.31 |
| m.dm | geriatrics | 45.14 | 43.09 |
| m.dm | Diagnosis | 43.82 | 36.94 |
| m.dm | Pre Illnesses (1) | 50.61 | 49.15 |
| m.dm | Pre Illnesses (2) | 61.11 | 61.56 |
| die.mnth | Socioeconomic Factors | 53.16 | 51.99 |
| die.mnth | internal.affairs | ! | ! |
| die.mnth | geriatrics | ! | ! |
| die.mnth | Diagnosis | 57.72 | 57.63 |
| die.mnth | Pre Illnesses (1) | 49.64 | 55.57 |
| die.mnth | Pre Illnesses (2) | 53.83 | 59.28 |

* “!” : A statistically significant model could not be developed.
