# Supplementary material for: A National Big Data Analysis on Alzheimer’s and Other Dementias in Türkiye
Source: J Aging Res. 2026 Feb 2;2026:1010110. doi: 10.1155/jare/1010110 (PMC12864165; doi:10.1155/jare/1010110)
Supplement: Supplementary file 1 — Supporting Information Additional supporting information can be found online in the Supporting Information section. [file JARE-2026-1010110-s001.zip › 4.Research_in_Context.docx]

**Research in Context**

**1. SYSTEMATIC REVIEW**

The review process includes a broad searching process for the knowledge on Alzheimer’s and other dementias in the literature. With this aim; PubMed, Web of Science and Scopus databases searched to reach related contents. Some of them are find useful and cited in the study by the authors. This process occurred from 01.01.2024 to 01.06.2024

**2. INTERPRETATION**

The main findings of the study presented below;

- The results obtained in the LR models show that being a foreigner and being in different social security categories has a positive effect on Alzheimer's.
- Being a woman increases the likelihood of diagnosis and shortens life expectancy.
- Being diagnosed in internal disease and geriatric clinics and the increase in the "intensive care unit" and "hospitalize" variables have a moderate or higher effect on diagnosis
- All pre-diseases either had no effect or had a minimal negative effect on all dependent variables.
- Individuals’ social and biological factors have insufficient explanatory power to explain the Alzheimer's/dementia diagnosis
- Findings reveal that contrary to popular belief, the assumption that AD/Dementia are diseases of old age should be re-examined.
- Being diagnosed with Alzheimer's disease increases by 49.11% for people who are not citizens of the Republic of Türkiye

**3. FUTURE DIRECTIONS**

Future directions of the study described below;

- The inability of various factors to explain Alzheimer's/dementia alone has highlighted the necessity of examining the factors that cause these diseases in a holistic manner.
- Alzheimer's disease is initiated by tau protein accumulation 20 years or more before the onset of symptoms suggests that there is an important timeframe
- Big data-based modeling, especially for technical analyzes regarding the diagnosis, treatment and prognosis of people with AD/Dementia in the preclinical process
- Understanding the pathogenesis of Alzheimer's disease (AD) depends on identifying how and to what extent an individual's life pattern interacts with it
- With the ability to predict the personalized pathogenic process of AD, innovative, data-driven interventions can be developed for prophylactic measures
